# Programme of the Atlanta Society of Medicine for 1905

**Published:** 1905-03

**Authors:** 


### SOCIETY REPORTS.

### PROGRAMME OF THE ATLANTA SOCIETY OF MEDICINE FOR 1905.

OFFICERS FOR 1905: President, Alex. W. Stirling; Vice-President, E. Bates Block; Secretary, W. B. Emery; Treasurer, E. Van Goidtsnoven.

CASE COMMITTEE: Claude A. Smith, Chairman; T. H. Hancock, C. E. Boynton.

EXECUTIVE COMMITTEE: T. V. Hubbard, Chairman; L. B. Clarke, C. W. Strickler.

PUBLICATION COMMITTEE: B. Wolff, Chairman; W. E. Wilmerding, W. B. Emery.

Meetings will be called to order punctually at 8 p.m., and, immediately after the consideration of applications for membership, cases, etc., will be demonstrated. The discussion of these cases will be immediately followed by the regular order of business, and will cease at 8:45 o'clock, unless the majority of the members present, by formal vote, desire it to continue.

Members will greatly assist the objects of the Society by informing the Chairman of the Case Committee, of all interesting cases which they can present to the Society. They are invited to present also macroscopical and microscopical specimens, instruments and apparatus.

All members are urgently requested to be as regular as possible in their attendanance at the meetings, and to participate in the discussions.

Papers read before the Society should not occupy more than twenty minutes each. By-law IV says that in discussing papers members must limit themselves to *ten* minutes, and speak only once upon each subject, "unless by special permission of the Society." By-law V says that during that part of the proceedings devoted to the Relation of Cases, no member shall be allowed to speak more than once in connection with any one case and then only for *five* minutes, without "special permission."

Article IV of the Constitution says: "All physicians in good standing, practicing in the county of Fulton, who are graduates of regular medical colleges, and no other persons, shall be eligible to active membership in this Society."

Article VII says: "Every member, except the Secretary and the Treasurer, shall pay into the treasury an annual contribution of two* dollars which shall be payable on the first day of January of each year, and if it is not paid by the first meeting in October of each year, he shall forfeit his membership, and his name shall be stricken from the roll, and of this he shall be notified by the Secretary. But the initiation fee of two dollars paid by new members shall include their dues for the remainder of the year."

Article VIII says: "Any member who in any way forfeits his membership in this Society, may be reinstated by formal application and ballot," and he (Article LI) "shall pay all arrearages up to date of dismissal and initiation fee."

Members are reminded of the necessity of having their names placed upon the Courthouse Register, without which they are disqualified as physicians in the eyes of the law.

Military surgeons and students of medicine are cordially invited to attend the meetings and to take part in the discussions.

PROGRAMME.

Jan. 5. President's Inaugural Address, "Our Primitive Ancestors—Anatomy *versus* Language," Dr. A. W. Stirling.

Jan. 19. (1) "The Doctor, the Child and the Community," Dr. Marion McH. Hull.

*Discussion:* Drs. Thrash, Fischer, Parks.

(2) Diseases of the Heart.

(*a*) Diagnosis, Dr. L. B. Clarke.

(*b*) The Action of Digitalis and Allied Drugs Upon the Vascular System, Dr. J. S. Todd.

*The question of raising the dues to $3.00 is to be brought up at the meeting on January 5th.

*Discussion:* Drs. Wilmerding, Armstrong, Van Goidtsnoven, Boynton.

(3) A New Method for the Sterilization and Chromatization of Catgut, Dr. C. R. Andrews.

Feb 2. (1) Prostatic, Vesicular and Urethral Massage, Dr. W. L. Champion.

*Discussion:* Drs. Olmsted, Emery, E. G. Jones, Ballinger, W. Goldsmith.

(2) Facts and Fallacies in Gastro-Intestinal Work, Dr. L. Amster.

*Discussion:* Drs. LeConte, Ashcroft, Block.

(3) Intestinal Indigestion—Its Dietetic and Rational Treatment, Dr. J. E. Ashcroft

*Discussion:* Drs. McRae, Riley, Gaines, Strickler.

Feb. 16. (*A*) Appendicitis.

(*a*) Causes, Dr. J. N. LeConte.

(*b*) Medical Treatment, Dr. J. B. Baird.

(*c*) Surgical Treatment, Dr. W. S. Elkin.

(*B*) Operations in Acute Abdominal Inflammations, Dr. B. F. Riley.

(*C*) The Present Status of Gastro-Intestinal Surgery, Dr. F. W. McRae.

*Discussion:* Drs. Nicolson, H. F. Harris, Strickler, Block, Hubbard.

Mar. 2. (1) (*a*) Mucous Collitis, Dr. Wm. Armstrong.

(*b*) Constipation, Dr. E. C. Thrash.

(*c*) Chronic Diarrhoea, Dr. J. N. LeConte.

*Discussion:* Drs. Elkin, H. F. Harris, Ashcraft, Baird, Amster, Rouglin.

(2) Smallpox—Diagnosis; Effects and Dangers of Vaccination, Dr. R. T. Dorsey, Jr.

*Discussion:* Drs. Todd and Baird.

Mar. 16. (1) The Necessity of Correcting Errors of Refraction, Dr. J. M. Crawford.

*Discussion:* Drs. W. E. Campbell, Hobbs, Wheeler.

(2) Report on Cases of Rheumatoid, Chronic Toxic and Osteo-Alteritis, Dr. M. Hoke.

*Discussion:* Drs. W. Goldsmith, Hancock, Willis Westmoreland, Elkin, Boynton, Claude Smith.

(3) Broncho-Pneumonia in Children, Dr. L. C. Rouglin.

*Discussion:* Drs. Samuels, Stephens, Boynton, L. B. Clarke.

* April 6. (1)

(*a*) Significance of Urinalysis in Pregnacy, Dr. E. G. Jones.

(*b*) Puerperal Eclampsia, Dr. Frances Bradley.

*Discussion:* Drs. L. M. Gaines, W. T. Jones, Earnest, Ellis, R. R. Kime, E. C. Davis.

(2) Some New Therapeutic Methods in Nervous Disorders, Dr. Wesley E. Taylor.

*Discussion:* Drs. Pinckney, Block, Brawner.

April 27. (1) Tumors of the Brain.

(*a*) Medical Aspect, Dr. E. B. Block.

(*b*) Surgical Aspect, Dr. W. P. Nicolson.

*Discussion:* Drs. W. Taylor, Brawner, Pinckney, Elkin, McRae.

(2) The Traumatic Neroses.

(*a*) General Review, Dr. T. V. Hubbard.

(*b*) Ocular and Aural Considerations, Dr. A. G. Hobbs.

*Discussion:* Drs. Todd, W. E. Campbell, King, Harris.

May 4. (1) Surgical Fever, Dr. Willis Westmoreland.

*Discussion:* Drs. J. L. Campbell, Barnett, Strickler, Earnest.

(2) The Physiological Factor in Diagnosis, Dr. J. C. Olmsted.

*Discussion:* Drs. E. G. Jones, B. F. Riley, Gaston, Thrash.

(3) Is Scarlet Fever a Scepticaemia? Dr. L. B. Clarke.

*Discussion:* Drs. Boynton, Visanski, Hynds, Samuels.

May 18. (1) The Anæmias, Dr. H. F. Harris.

*Discussion:* Drs. Strickler, Claude Smith, Wilmerding, Block, Fischer.

---

* The second April meeting is postponed one week on account of the meeting of the State Society.

(2) Abortion, Criminal and Legitimate, Dr. B. F. Riley.
*Discussion:* Drs. Armstrong, W. Jones, Fischer.
(3) Vaginal Drainage for Pelvic Abscess in Cases Necessitating Abdominal Section, Dr. E. C. Davis.
*Discussion:* Drs. Kime, Ellis, Earnest.

June 1. (1) Surgery of the Testicle, Dr. Wm. S. Goldsmith.
*Discussion:* Drs. Gaines, Strickler, Fowler.
(2) (*a*) Some Genito-Urinary Cases, Dr. W. B. Emery.
(*b*) Cystitis, Dr. E. G. Ballenger.
*Discussion:* Drs. Champion, Olmsted, E. G. Jones.
(3) Prophylaxis of Veneral Disease, Dr. L. C. Rouglin.
*Discussion:* Drs. Samuels, L. B. Clarke.

June 15. (1) The Examiner in Life Insurance, Dr. W. E. Wilmerding.
*Discussion:* Drs. Todd, White.
(2) The Treatment of Serious Accidents, Dr. C. W. Strickler.
*Discussion:* Drs. Elkin, Willis Westmoreland, Hancock.
(3) The McGraw Ligature and the Murphy Button as they originated from Gaston's Operation, Dr. J. McFadden Gaston.
*Discussion:* Drs. McRae, Kime, Davis.

July 6. (1) (*a*) The Uses of Water in Medicine, Dr. J. S. Todd.
(*b*) The Uses of Electricity in Medicine, Dr. B. Wolff.
(*c*) The Uses of Exercise in Medicine, Dr. Th. Toepel.
*Discussion:* Drs. Hutchins, Duncan, Claude Smith, Brawner.
(2) Treatment of Ulcers, Dr. A. L. Fowler.
*Discussion:* Drs. Hutchins, J. L. Campbell, Ward.

July 20. (1) Post-Operative Treatment, Dr. C. P. Ward.
*Discussion:* Drs. A. L. Fowler, Gaines, Barnett.

(2) Life History of the Typhoid Bacillus, Dr. E. B. Block.
*Discussion:* Drs. H. F. Harris, Claude Smith.
(3) Disseminated Sclerosis, Dr. J. Creston King.
*Discussion:* Drs. Pinckney and Taylor.

Aug. 3. (1) Surgery of the Gall Bladder, Dr. J. L. Campbell.
*Discussion:* Drs. C. Strickler, Elkin, J. McFadden Gaston.
(2) Haemorrhoids, Dr. L. M. Gaines.
*Discussion:* Drs. Todd, Duncan, McRae, Champion, Amster.
(3) Dogmas and Fashions in Practice, Dr. E. C. Cartledge.
*Discussion:* Drs. Parks and White.

Aug. 17. (1) Cancer.
(*a*) Prevention, Dr. Claude Smith.
(*b*) Surgical Treatment, Dr. M. B. Hutchins.
*Discussion:* Drs. H. F. Harris, Nicolson, Armstrong.
(2) Empyema, Dr. S. T. Barnett.
*Discussion:* Drs. W. Goldsmith, Gaston, J. L. Campbell.

Sept. 7. (1) Hay Fever. Dr. E. Van Goidtsnoven.
*Discussion:* Drs. Hobbs, Wheeler, Brawner.
(2) (*a*) Electricity in Diseases of the Stomach, Dr. J. E. Ashcraft.
(*b*) Œsophagoscopy, with Demonstration, Dr. L. Amster.
*Discussion:* Dr. LeConte.
(3) Surgery of Tubercular Adenitis, Dr. L. M. Gaines.
*Discussion:* Drs. Nicolson, Elkin, Armstrong, Boland.

Sept. 21. (1) Opium.
(*a*) Its Uses, Dr. J. S. Todd.
(*b*) State Provision for Drug Habitues, Dr. C. C. Stockard.
*Discussion:* Drs. Davis, Andrews, Earnest.

(2) Tetanus and the Public, Dr. S. T. Barnett.
*Discussion*: Drs. Baird, Pinckney, Block.
(3) Rheumatism in Children, Dr. L. P. Stephens.
*Discussion:* Drs. Visanski, Boynton, Van Goidtsnoven, McH. Hull.

Oct. 5. (1) Diet.
(*a*) In Health and as a Cause of Disease, Dr. R. R. Kime.
(*b*) In Disease, Dr. J. S. Todd.
*Discussion:* Drs. H. F. Harris, Strickler, Wolff.
(2) (*a*) Demonstration of Methods of Regional Diagnosis in Diseases of the Brain, Dr. W. E. Taylor.
(*b*) Hysteria, Dr. S. G. C. Pinckney.
*Discussion:* Drs. Block, Brawner.

Oct. 19. (1) Pancreatitis, Dr. H. F. Harris.
*Discussion:* Drs. C. Strickler, Amster, Ashcraft.
(2) Nasal and Post-Nasal Work, Dr. A. E. Wheeler.
*Discussion:* Dr. Hobbs.
(3) Treatment of Ear Affections, Dr. J. M. Crawford.
*Discussion:* Dr. Campbell.

Nov. 2. (1) Surgical Treatment of Movable Kidney, Dr. E. C. Davis.
*Discussion:* Drs. Elkin, Nicolson, W. Goldsmith.
(2) Drainage After Laparotomy, Dr. James N. Ellis.
*Discussion:* Drs. W. Jones, McRae, Riley
(3) Treatment of Infection After Labor and Miscarriage, Dr. J. L. Campbell.
*Discussion:* Drs. Earnest, Kime, M. G. Campbell.

Nov. 16. (1) Neurasthenia, Dr. E. B. Block.
*Discussion:* Drs. Wolf, E. Van Goidtsnoven, Brawner, Pinckney, Thrash.
(2) Lumbago and Sciatica—Pathology and Treatment, Dr. Frank Boland.
*Discussion:* Drs. Ward, Ashcraft, Todd.

(3) The Disposal of the Dead, Dr. R. Hynds.
*Discussion:* Drs. Wolff, Thrash.

Dec. 7. (1) Railway Spine, Dr. W. P. Nicolson.
*Discussion:* Drs. Hancock, Strickler.

(2) Fracture at the Elbow Joint, Dr. Willis Westmoreland.
*Discussion:* Drs. Hoke, Boland, Goldsmith.

(3) Renal Diseases.
(*a*) Prevention, Dr. W. Jones.
(*b*) Prognosis, Dr. Claude Smith.
(*c*) Some Observations, Dr. R. Kime.
*Discussion:* Drs. Wilmerding, Todd.

Dec. 21. (*a*) Annual Message of the Retiring President, Dr. A. W. Stirling.
(*b*) Election of Officers.

